# *Candida albicans* biofilm extracellular vesicles deliver candidalysin to epithelial cell membranes and induce host cell responses

**DOI:** 10.1128/iai.00404-24

**Published:** 2025-04-02

**Authors:** Sejeong Lee, Antzela Tsavou, Robert Zarnowski, Rita Pforte, Stefanie Allert, Thomas Krüger, Olaf Kniemeyer, Axel A. Brakhage, Tam T. T. Bui, David R. Andes, Jonathan P. Richardson, Bernhard Hube, Julian R. Naglik

**Affiliations:** 1 Centre for Host-Microbiome Interactions, Faculty of Dentistry, Oral & Craniofacial Sciences, King’s College London 61139 , London, United Kingdom; 2 Department of Medicine, Infectious Disease Division, School of Medicine & Public Health, University of Wisconsin-Madison 200889 , Madison, Wisconsin, USA; 3 Department of Microbial Pathogenicity Mechanisms, Leibniz Institute for Natural Product Research and Infection Biology-Hans-Knöll Institute 28406 , Jena, Thuringia, Germany; 4 Department of Molecular and Applied Microbiology, Leibniz Institute for Natural Product Research and Infection Biology-Hans-Knöll-Institute 28406 , Jena, Thuringia, Germany; 5 Institute of Microbiology, Friedrich Schiller University 163335 , Jena, Thuringia, Germany; 6 Centre for Biomolecular Spectroscopy and Randall Division of Cell and Molecular Biophysics, King’s College London 105716 , London, United Kingdom; NIAID, NIH, Washington, DC, USA

**Keywords:** *Candida albicans*, candidalysin, extracellular vesicles, fungal infection, host-pathogen interactions, cell membranes, membrane transport, host response

## Abstract

Extracellular vesicles (EVs) are heterogeneous particles encapsulated with a phospholipid bilayer membrane. EVs have evolved diverse biological functions, serving mainly as prominent mediators and regulators of cell-cell communication. This study investigated whether candidalysin, a key virulence factor in *Candida albicans* infections, is present within EVs derived from *C. albicans* biofilms and retains activity by inducing host immune responses. We found that biofilm EVs contain candidalysin and can permeabilize planar lipid bilayer membranes in a dose-dependent manner. However, biofilm EVs were unable to damage oral epithelial cells (OECs) but were able to induce cytokine responses. Notably, EVs obtained from biofilms cultured for 24 h and 48 h exhibited differences in cargo composition and their ability to activate OECs. This study highlights the potential of biofilm EVs as a toxin delivery system during *C. albicans* infection and identifies temporal differences in the ability of EVs to activate epithelial cells.

## INTRODUCTION

Intercellular communication is mediated by several types of molecules including ions, metabolites, secreted proteins, receptors, and membranous particles. These cell-cell communications are important for regulating cellular development, tissue homeostasis, and immune interactions in disease (1). During host-pathogen interactions, communicable substances are transported to host cells, causing altered cellular activities and triggering immune activation. One type of membranous particle involved in intercellular communication is extracellular vesicles (EV). EVs are heterogeneous microparticles produced by all kingdoms of life, which contain cargo embedded and enclosed within a lipid bilayer membrane. Thus, EVs can serve as export or delivery systems (2, 3).

Gram-negative bacteria use specialized EVs known as outer membrane vesicles (OMVs) as a toxin delivery system (4). OMVs can carry various cargo including virulence factors, nucleic acids, and antigens. Similarly, microbial toxins that lack an export signal such as pneumolysin and cytolysin A are released from microbes by vesicle-mediated delivery (5, 6). Previously, it was thought that the production of EVs by Gram-positive bacteria, mycobacteria, and fungi may not occur due to their thick cell walls, but several studies have shown that these organisms also produce EVs (7–9). Indeed, more than 20 species of fungi are known to produce EVs, including the common human pathobiont *Candida albicans* (10–12). Recently, EVs from planktonic and biofilm cultures of *C. albicans* were detected by scanning electron microscopy, highlighting the functional importance of matrix materials. Interestingly, yeast-derived EVs were found to inhibit biofilm formation (13), whereas biofilm-derived EVs confer drug resistance (14).

Candidalysin is encoded by the extent of cell elongation 1 gene (*ECE1*) and is a key virulence factor for *C. albicans* infection (15, 16). During mucosal infection, candidalysin is secreted from hyphae into an invasion pocket, where it causes cell damage and immune activation in a dose-dependent manner (15, 17, 18). Candidalysin activates the epidermal growth factor receptor, leading to the activation of mitogen-activated protein kinase (MAPK) signaling pathways, MAPK phosphatase 1, and the upregulation of the transcription factor c-Fos, which drives cytokine production (18–21). However, the relationship between the presence of key virulence factors in *C. albicans* EVs and their role in pathogenesis is unclear. Recent investigations of EVs produced by *C. albicans* biofilms (which consist predominantly of hyphae) revealed the presence of Ece1p (the parental protein of candidalysin) after trypsin digest (14, 22), suggesting that *C. albicans* EVs may act as a toxin delivery system. However, it remains unclear whether mature candidalysin is present in EVs and how the candidalysin-containing EVs exert biological functions in comparison with free candidalysin secreted from hyphae.

Here, we investigated whether *C. albicans* EVs contain candidalysin and whether these EVs function as an effective delivery system for candidalysin to reach the host epithelium. We used EVs produced from *C. albicans* biofilms and characterized their membrane permeabilization activities. Notably, the application of biofilm-derived EVs to epithelial cells activated innate immune responses in a candidalysin-dependent and -independent manner, depending on the maturity of the EVs. Collectively, these findings suggest that EVs from *C. albicans* biofilms can act as a toxin delivery system and demonstrate that epithelial cells are immunologically responsive to candidalysin-containing EVs.

## RESULTS

### Candidalysin is present in extracellular vesicles from *C. albicans* biofilms

**Fig 1:**
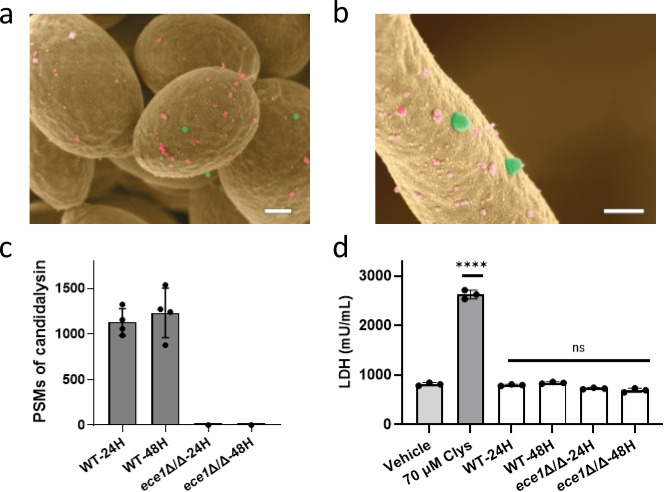
Candidalysin-containing EVs are unable to cause cellular damage. A scanning electron microscopy (SEM) image of EVs (green) and extracellular matrix (pink) on the surface of *C. albicans* growing as yeast (**a**) and biofilm (**b**). Scale bars represent 200 nm. (**c**) Liquid chromatography-tandem mass spectrometry (LC-MS/MS) analysis of EVs obtained from *C. albicans* WT and *ece1*Δ/Δ biofilms cultured for 24 h and 48 h. Average peptide spectrum match (PSM) values (*n* = 4 biological repeats from WT-EVs and *n* = 1 from *ece1*Δ/Δ-EVs) are shown with standard deviation. No candidalysin was detected from *ece1*Δ/Δ-EVs. (**d**) Epithelial cell damage was monitored with biofilm EVs. WT-24H, WT-48H, *ece1*Δ/Δ-24H, and *ece1*Δ/Δ-48H EVs of 3 × 10^12^ p/mL were applied to oral epithelial cells for 24 h and lactate dehydrogenase activity was quantified as a marker of cellular damage. As a positive control, epithelial cells were treated with candidalysin peptides of 70 µM. Data are shown as the mean ± standard deviation of *n* = 3 biological repeats. Statistical significance was calculated by unpaired *t*-test compared to vehicle-treated cells (*P* **** <0.0001).

To determine whether candidalysin is present in *C. albicans* EVs, we isolated EVs from WT strain SN250 (23) grown for 24 h and 48 h (WT-24H; WT-48H). These time points were chosen as EV production becomes saturated by 48 h after biofilm initiation (14). The presence of EV-like structures was confirmed by electron microscopy (Fig. 1a and b) and EV-specific markers (Hse1 and Vps27) were found in *C. albicans* EVs (14). The size of the isolated *C. albicans* EVs was approximately 150–200 nm in diameter (Fig. S1), which is noticeably larger than exosome particles (24).

Next, we used liquid chromatography-tandem mass spectrometry (LC-MS/MS) to analyze the molecular cargo present in EVs derived from *C. albicans* biofilms. The method was optimized for the unambiguous determination of candidalysin and all other *ECE1*-derived peptides. It enables the clear distinction between mature candidalysin (ending with lysine; K) and the immature toxin (ending with lysine-arginine; KR) by evaluating the resulting tandem mass spectra based on the Sequest database search algorithm (15, 25). Notably, high numbers of peptide spectrum matches (PSMs) corresponding to the presence of mature candidalysin were observed in EVs obtained from biofilms cultured for 24 h and 48 h (Fig. 1c). In contrast, EVs obtained from a *C. albicans* mutant that was unable to produce candidalysin, as the encoding *ECE1* gene was deleted, (*ece1*Δ/Δ-24H and *ece1*Δ/Δ-48H; *C. albicans ece1*Δ/Δ strain was created as described previously [15]) revealed no or little PSM values. These data demonstrate that candidalysin is present in EVs obtained from *C. albicans* biofilms.

### Candidalysin-containing EVs do not damage human oral epithelial cells *in vitro*

Since candidalysin is currently the only known *C. albicans* factor that directly induces epithelial cell damage (15), we tested whether candidalysin-containing EVs could also damage epithelial cells *in vitro* using lactate dehydrogenase activity as a marker of cellular damage. TR146 oral epithelial cells (OECs) were treated with EVs at the final concentration of 3 × 10^12^ particles/mL (p/mL) obtained from biofilms cultured for 24 h (WT-24H) and 48 h (WT-48H), and exhausted culture medium was collected after 24 h and assayed. However, no damage was observed. Biofilm EVs lacking candidalysin (*ece1*Δ/Δ-24H and *ece1*Δ/Δ-48H) also did not induce cell damage (Fig. 1d). This implies that (i) candidalysin in EVs is not functional and/or (ii) insufficient amounts of candidalysin are delivered to OECs to cause damage *in vitro* under the conditions tested. We also tested the effects after 48 h incubation; however, no cell damage was observed (data not shown).

### Candidalysin-containing EVs induce membrane fusion and permeabilization on artificial membranes

**Fig 2:**
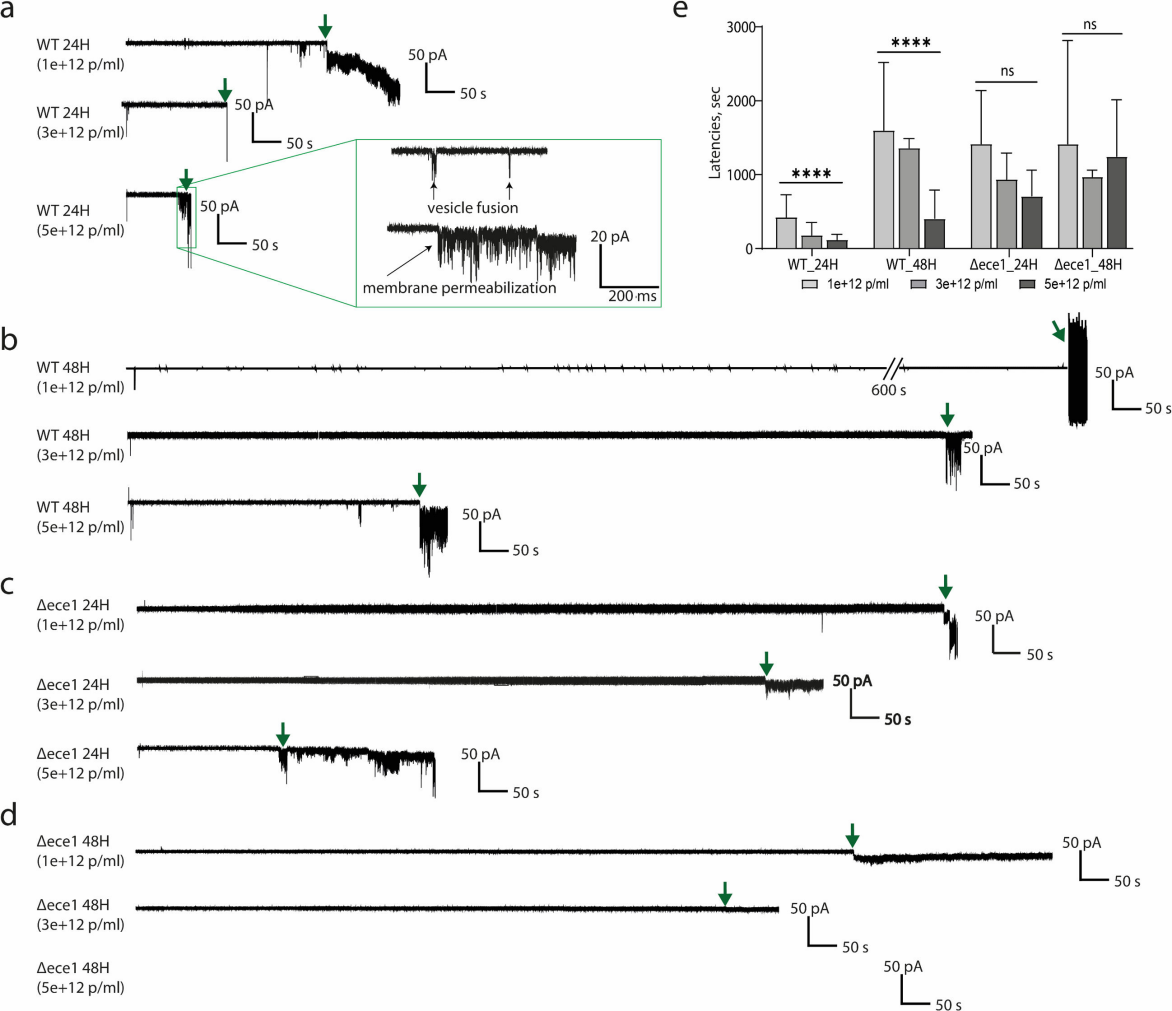
Candidalysin-containing EVs permeabilize DPhPC bilayer membranes in a concentration-dependent manner. Changes in electrical current across a DPhPC bilayer were monitored following the addition of (**a**) WT-24H EVs, (**b**) WT-48H EVs, (**c**) *ece1*Δ/Δ-24H EVs, and (**d**) *ece1*Δ/Δ-48H EVs (1 × 10^12^, 3 × 10^12^, and 5 × 10^12^ p/mL). Vesicle fusion and membrane permeabilization were monitored (see zoomed-in box). (**e**) The membrane permeabilization kinetics, defined as the latency until membrane permeabilization, are presented as a bar chart (see Table S1). Concentration-dependent membrane permeabilization was observed based on data from at least six independent experiments. Mean values of latency and their corresponding standard deviations are displayed. Statistical significance was determined using a one-way analysis of variance (*P* **** <0.0001, ns = non-significant).

To further investigate the functionality of candidalysin from biofilm EVs, we employed a more sensitive approach. We monitored the membrane fusion and membrane permeabilizing potential of EVs when applied to zwitterionic lipid bilayers comprising 1,2-diphytanoyl-sn-glycero-3-phosphocholine (DPhPC) lipids. Previous studies showed that the application of synthetic candidalysin to DPhPC lipid bilayers induces membrane permeabilization (18). Additionally, the kinetics of this permeabilization correlate with cellular damage potential and are relative to the concentration of candidalysin (18). Therefore, EVs at the final concentration of 1 × 10^12^, 3 × 10^12^, and 5 × 10^12^ p/mL were added to the bilayers, and electrical current changes were monitored over 30 min at an applied potential of −50 mV, a physiologically relevant membrane potential across the human cellular membrane (26). Upon interaction of EVs with bilayers, two types of current changes were observed: reversible short-lived peaks and permanent current drops, implying membrane fusion and permeabilization, respectively (Fig. 2a).

Membrane permeabilization kinetics were monitored by measuring latency values defined as the time period from the application of EVs to the DPhPC lipid bilayer until current changes were observed. Kinetic analysis revealed that both WT-24H and WT-48H EVs caused membrane permeabilization in a concentration-dependent manner (Table S2). Specifically, WT-24H EVs at 5 × 10^12^ p/mL induced membrane permeabilization four times faster than those at 1 × 10^12^ p/mL (120 ± 70 s vs 430 ± 330 s, *P*-value < 0.005) (Fig. 2a and e). Similarly, WT-48H EVs exhibited a fourfold slower permeabilization kinetics compared to WT-24H EVs (1,950 ± 570 s vs 420 ± 390 s, *P*-value < 0.005) (Fig. 2b and e).

Our previous work demonstrated that *C. albicans* candidalysin induces membrane permeabilization in a concentration-dependent manner (18). By comparing latency values to those of synthetic candidalysin (18), approximately 2 µM candidalysin was estimated to be present in WT-24H EVs at 1 × 10^12^ p/mL, while approximately 0.5 µM was estimated to be present in WT-48H EVs at the same concentration.

In contrast, candidalysin-deficient EVs (*ece1*Δ/Δ-EVs) exhibited concentration-independent membrane permeabilization (Fig. 2c through e), suggesting relatively weak or non-specific interactions with DPhPC lipid bilayers. This data demonstrates that candidalysin present in EVs from WT *C. albicans* biofilms can cause concentration-dependent bilayer permeabilization.

### Candidalysin-containing EVs obtained from 24 h and 48 h induce cytokine production in a candidalysin-dependent and candidalysin-independent manner

While the application of WT EVs to OECs failed to cause cellular damage, we hypothesized that a sufficient concentration of candidalysin may still be present to induce innate immune responses. Candidalysin activates OECs, leading to the production of immuno-modulatory cytokines (15, 21, 27–31). To determine whether candidalysin-containing EVs could also activate similar epithelial responses, EVs (3 × 10^12^ p/mL; equivalent to ~6 µM candidalysin) were applied to epithelial cells, and cytokine production was monitored.

**Fig 3:**
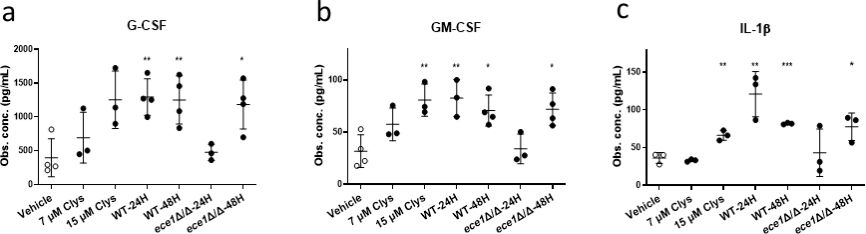
EVs from *C. albicans* biofilms induce cytokine secretion. Oral epithelial cells were exposed to *C. albicans* biofilm EVs for 24 h, and secretion of cytokines was quantified. Observed concentrations (Obs. conc.; pg/mL) are shown. Treatments with synthetic candidalysin peptide (7 µM and 15 µM) were included as a positive control. WT-24H EVs, WT-48H EVs, and *ece1*Δ/Δ-48H were able to induce some cytokine release including (**a**) G-CSF, (**b**) GM-CSF, and (**c**) IL-1β. Data are shown as the mean ± standard deviation of *n* = 3–4 biological repeats. Statistical significance was calculated using an unpaired two-tailed *t*-test in comparison with the vehicle (*P* * <0.05, *P* ** <0.01, and *P* *** <0.001).

Both 24H and 48H EVs were capable of inducing significant amounts of G-CSF, GM-CSF, and IL-1β secretion (equivalent to 15 µM synthetic candidalysin) (Fig. 3a through c). In contrast, *ece1*Δ/Δ-24H EVs failed to induce cytokine responses, indicating that candidalysin in EVs can induce cytokine production. However, *ece1*Δ/Δ-48H EVs were able to induce similar significant levels of cytokine responses compared to WT-48H EVs, suggesting that the biofilm membranes of 48H EVs may trigger cytokine production independently of candidalysin.

### Candidalysin-containing EVs obtained from 24 h and 48 h show different cargo and different detergent susceptibility

While WT-24H EVs and WT-48H EVs contained near-identical PSMs for candidalysin, they displayed different membrane permeabilization kinetics (Fig. 2) and distinct cytokine responses in a candidalysin-dependent and candidalysin-independent manner, respectively (Fig. 3). We hypothesized that these distinct responses may be driven by differences in the composition of molecular cargo present in 24 h and 48 h EVs. To test this, we compared cargo proteomes by tandem mass spectrometry (LC-MS/MS), which revealed 59 proteins that were exclusively present in 24H-EV and associated with hyphal growth or membrane transport (Table S3). These included proteins for hypha-related growth, fatty acid synthase, a core subunit of protein-conducting channel Sec61, a subunit of the translocase of mitochondrial outer membrane complex, and a subunit of ion transporter complex.

**Fig 4:**
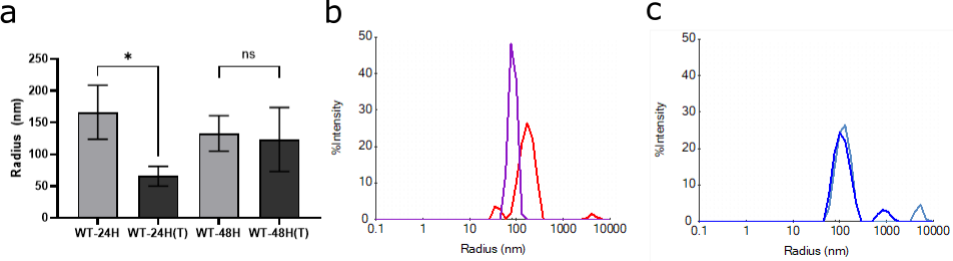
Candidalysin-containing EVs exhibit differences in detergent susceptibility. The radius of EVs obtained from wild-type *C. albicans* biofilms cultured for 24 h and 48 h was monitored in response to treatment with Triton X-100 using dynamic light scattering. (**a**) The bar chart represents the mean ± standard deviation of *n* = 3 independent experiments. Statistical analysis was performed using an unpaired two-tailed *t*-test (*P* * <0.05, ns = non-significant). (**b**) Hydrodynamic radius distribution by intensity for WT-24H EVs without (red) and with Triton treatment (purple). The reduction in radius after detergent treatment indicates membrane-ruptured EVs. (**c**) Similar analysis of WT-48H EVs, without (light blue) and with Triton treatment (blue) shows that the radius of WT-48H EVs remained unchanged, indicating the resistance to detergent-induced membrane rupture.

Next, we hypothesized that differences in EV membrane composition at 24 h vs 48 h might also affect the membrane permeabilization activity of candidalysin-containing EVs. To test this hypothesis, we monitored the detergent susceptibility of 24 h and 48 h EVs by measuring the EV radius after treatment with 0.1% Triton X-100 for 30 min using dynamic light scattering (DLS) (Fig. 4). The radius of WT-24H EVs decreased from 170 ± 40 nm to 70 ± 20 nm (*P*-value < 0.05), indicating membrane rupture in response to the detergent, while the radius of WT-48H EVs remained unchanged with increased fluctuations from 130 ± 30 nm to 120 ± 50 nm, indicating resistance. To confirm whether this discrepancy was due to membrane differences, we tested candidalysin-deficient EVs (*ece1*Δ/Δ-48H), which exhibited no changes in radius after treatment with 0.1% Triton X-100 (Fig. S1). These findings strongly imply that 24H-EVs and 48H-EVs have different detergent tolerances, likely due to their distinct lipid compositions.

**Fig 5:**
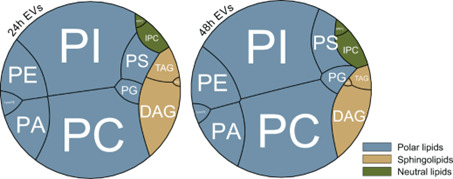
Comparative lipidomics of *C. albicans* biofilm EVs. The circular Voronoi treemaps reflect absolute amounts of distinct lipid classes detected in EVs isolated from 24 h and 48 h old biofilms, respectively. The coloration of individual clusters is based on their classification and shows polar lipids in gray-blue, sphingolipids in rainforest green, and neutral lipids in putty yellow. Data shown are the mean of *n* = 7 independent experiments. Polar lipids: PA, phosphatidic acid; PC, phosphatidylcholine; PE, phosphoethanolamine; PG, phosphatidylglycerol; PI, phosphoinositol; lysoPC, lyso phosphatidylcholine; lysoPE, lysophosphoethanolamine; PS, phosphatidylserine. Sphingolipids: IPC, inositol-P-ceramides; MIPC, mannose-inositol-P-ceramides; MI(IP)_2_C, mannosyl-di-(inositolphosphoryl)-ceramide. Neutral lipids: DAG, diacylglycerols; TAG, triacylglycerols; ERG, ergosterol esters.

Furthermore, our lipidomic analysis detected quantitative differences in certain lipids with ergosterol (ERG), diacylglycerol (DAG), and triacylglycerol (TAG) being more abundant in 24H-EVs (Fig. 5; Table S4). It is known that ERG, DAG, and TAG are crucial lipid elements for regulating membrane mobility (32), suggesting different membrane permeability properties between EVs of 24 h and 48 h.

Collectively, these data indicate that (i) less mature biofilm EVs (i.e., 24 h) potentially facilitate candidalysin’s delivery to the OEC membrane to induce cytokine production, and (ii) mature biofilm EVs (i.e., 48 h), while harboring candidalysin, activate OEC cytokine production in a candidalysin-independent manner. This is likely due to the different cargo composition of 24 h and 48 h EV membranes as indicated by DLS and proteomic/lipidomic analysis (Fig. 4 and 5; Table S3 and S4), further confirming that 24 h and 48 h EVs appear functionally distinct.

## DISCUSSION

Bacterial, fungal, and parasitic pathogens produce EVs containing virulence factors that interact with host cells and promote disease progression (33–37). These virulence factors include pore-forming toxins, lipids, enzymes, and extracellular matrix, which can enhance invasion into host cells and modulate host immune responses (7, 38–41). At mucosal surfaces, the full pathogenic potential of *C. albicans* is achieved when candidalysin is secreted from invasive hyphae and accumulates in the invasion pocket where it damages epithelial membranes (17). In this study, we investigated whether candidalysin is present in EVs produced by *C. albicans* and whether EV-derived candidalysin exhibits damaging potential to OECs and modulates host immune responses. Our findings reveal that candidalysin is present in biofilm-derived EVs and is capable of inducing permeabilization of artificial membranes and modest levels of immune activation in OECs. However, candidalysin-containing EVs did not cause OEC damage, suggesting that insufficient concentrations of candidalysin were present or delivered to the epithelial cell membrane. Additionally, our findings suggest that the maturity of biofilm EVs may play a crucial role in determining whether EVs activate OEC responses in a candidalysin-dependent or -independent manner. Taken together, our study highlights the multifunctional role of EVs, which can serve as a toxin delivery system during *C. albicans* infection *in vitro*.

Two studies have shown the presence of Ece1p (parent protein of candidalysin) in biofilm EVs (14, 22). However, in both cases, the analysis was performed after trypsin digest, which cleaves at the same sites as kexin proteinases; the enzymes within *C. albicans* that process full-length Ece1p into several peptides including candidalysin (15, 25). Therefore, it was unclear whether candidalysin was present in EVs at the point of EV production. As such, this is the first study to directly show the presence of candidalysin in *C. albicans* EVs. As candidalysin is a hypha-associated factor, the toxin was only observed in biofilm-derived EVs consisting predominantly of hyphae.

Due to biological heterogeneity, identifying physiological concentrations of EVs, as well as the actual concentration of candidalysin present in EVs, presents a challenge. In this study, we used a high concentration of EVs (3 × 10^12^ p/mL) to measure their cell-damaging potential, containing approximately ~6 µM of candidalysin. While candidalysin-containing EVs were capable of permeabilizing artificial membranes, they were incapable of causing damage to OECs. However, candidalysin-containing EVs were able to induce cytokine secretion. One possible explanation is that the concentration of active candidalysin in EVs is low, which is insufficient to cause OEC damage (the minimum concentration of candidalysin required to cause cellular damage after 24 h when applied to OECs *in vitro* is ~15 µM) (15, 18). Another possibility is that the function of candidalysin is influenced by its conformation and specific location within EVs. Due to its hydrophobic nature, it is likely that candidalysin is encapsulated within EVs rather than being exposed on the outside, requiring the disruption of EVs and/or conformational changes for full activity. A recent study indicated that candidalysin’s activity is modulated by the adjacent peptide fragments within the parental protein Ece1p (42), suggesting that candidalysin undergoes a conformational change to transition from a protected state to an exposed, active state. Additionally, it is noteworthy that EVs containing the bacterial toxin pneumolysin exhibit no cytotoxic effects on epithelial cells (43), suggesting that the uptake of EVs and delivery of microbial toxins to host cells may not necessarily dictate the levels of cytotoxicity.

Candidalysin-containing biofilm EVs of differing maturity (24 h vs 48 h) exhibited different biological activity when compared to candidalysin-deficient biofilm EVs. For instance, while both candidalysin-containing EVs (24 h and 48 h) demonstrated a similar level of cytokine production, EVs isolated at 24 h induced cytokine production in a candidalysin-dependent manner. In contrast, EVs isolated at 48 h induced cytokine production in a candidalysin-independent manner. The differences in functionality observed between 24 h and 48 h EVs could be explained by (i) variations in protein cargos, including the presence of different proteins or differing concentrations of the same proteins, and (ii) differences in lipid composition resulting in different membrane properties. The unique proteins detected in 24H-EVs serve diverse functions including cellular metabolism, oxidation, fatty acid chain elongation, sphingolipid biosynthesis, protein biosynthesis, and the transport of ions and proteins, indicating essential physiological roles in the growth and maturation of EVs. Sphingolipids, especially sphingomyelin, are recognized for enhancing vesicle internalization via raft pathways (44, 45), potentially increasing the membrane fluidity of 24H-EVs. Additionally, the presence of ion transporters and proton pumps is likely to increase membrane permeability by facilitating the movement of ions and proteins across the membrane. Previous studies have shown significant differences in cargo composition between planktonic and biofilm EVs and their function in sharing community resources such as biofilm biogenesis, yeast-to-hyphae morphogenesis, and extracellular matrix formation (13, 14, 22).

Quantitative variations in lipid composition were observed, with 24H-EVs exhibiting higher levels of ergosterol and glycerol (DAG, TAG), thus confirming differences in the physical properties of EV membranes isolated at different time points. Previous studies reported the impact of sterols and glycerol on membrane stability and permeability, suggesting that the decreased levels of ergosterol contribute to reduced membrane fluidity (46). Additionally, studies on glycerol-deficient *Candida* have shown defects in invasion and membrane permeabilization *in vitro* (47), which aligns with our findings of increased membrane permeability in 24H-EVs. This likely explains why permeabilization of DPhPC bilayers by 24H-EVs was more efficient than 48H-EVs, even though the candidalysin PSM values were similar. The more efficient membrane fusion and permeabilization activities suggest a higher degree of lipid fluidity (48). This hypothesis is supported by the DLS analysis, which demonstrated that 24H-EVs were more sensitive to detergent-induced membrane permeabilization. Conversely, 48H-EVs were less sensitive to detergent, thereby suggesting a lower degree of lipid fluidity.

In summary, this study demonstrates that candidalysin is present in EVs and can be delivered to epithelial cells and that the efficiency of delivery is influenced by EV maturity and membrane fluidity. When placed in the context of a *C. albicans* infection, we hypothesize that candidalysin is secreted from hyphae into an invasion pocket in both a “free” form (high levels; as previously shown [17]) and in EVs (low levels). Both forms will likely have different functions, with “free” candidalysin concentrated within the invasion pocket, directly targeting the OEC membrane to induce cell damage/lysis to potentially access nutrients, and EV-delivered candidalysin, which potentially has several “non-damaging” functions. One intriguing possible function is that (because candidalysin delivery depends on maturity and membrane properties) EV delivery of candidalysin may function to induce “host cell priming” that benefits the fungus in a manner that is currently unknown. Likewise, epithelial cells may sense secreted EVs to optimize the host response to *C. albicans* infection.

## MATERIALS AND METHODS

### Preparation of *Candida albicans* extracellular vesicles

*C. albicans* SN250 EVs (SC5314 derivative) were isolated as described by Zarnowski et al. (40). Briefly, *C. albicans* biofilm cultures were grown in 2.2 L large-scale polystyrene roller bottles at 37°C/4 rpm for 24 h and 48 h. After incubation, the culture medium was carefully decanted, and any suspended debris was removed by filtration through a paper filter, followed by filter sterilization. The collected culture supernatants were concentrated to 25 mL using two coupled Vivaflow 200 units (Sartorius AG) equipped with Hydrosart 30 kDa cut-off membranes. To eliminate larger cellular or membrane-like particulates, samples were centrifuged at 10,000 × *g* for 1 h at 4°C, followed by ultracentrifugation in a Beckman Coulter Optima MAX-XP Ultracentrifuge equipped with an MLA-50 fixed rotor at 100,000 × *g* for 1.5 h at 4°C. The resulting supernatants were discarded, and the pellets containing the EVs were resuspended in 5 mL of phosphate-buffered saline (PBS, pH 7.2). The resuspended samples underwent a second ultracentrifugation step using an MLS-50 swinging bucket rotor at 100,000 × *g* for 1 h at 4°C. The collected EVs were further purified and subjected to flash size-exclusion chromatography using qEV/35 nm columns (Izon Science). After purification, the samples were filter sterilized and stored at 4°C until further use.

### Characterization of EVs using nanoparticle tracking

The purified EVs were quantified using nanoparticle tracking analysis. To ensure accurate measurements, a 0.2 µm filter pore was used, and samples were diluted in PBS to a final volume of 1 mL (14). Pre-testing was conducted to determine the optimal particle count of 30–100 particles per frame rate. Measurements were performed using a NanoSight NS300 (Malvern) system equipped with a sample assistant autosampler. The following settings were applied: the camera level was increased to 16, and the camera gain was set to 2 to optimize the visibility of nanoparticles without saturating the particle signal. Each measurement consisted of six 1-min-long videos, with a 5 s delay between the sample introduction and the start of the first measurement. For the detection threshold analysis, the particle counts were limited to 10–100 red crosses and no more than 5–7 blue crosses. The acquired data were analyzed using the NanoSight Software NTA 3.4 Build 3.4.003 (Malvern). To ensure reliable results and minimize data bias caused by single large particles, at least 1,000 events were tracked per sample.

### Scanning electron microscopy imaging of EVs

The surface of *C. albicans* biofilms grown in 6-well plates was imaged using scanning electron microscopy (SEM) as previously described (14). Briefly, 40 µL of an inoculum containing 108 cells/mL in RPMI medium was added to the coverslips and incubated at 37°C for 60 min. After incubation, 1 mL of RPMI medium was added, and the plates were further incubated at 37°C for 20 h. Following incubation, 1 mL of fixative (composed of 4% formaldehyde and 1% glutaraldehyde in PBS) was added, and the plates were incubated at 4°C overnight. The coverslips were subsequently washed with PBS and incubated in 1% osmium tetroxide (OsO_4_) for 30 min to enhance contrast. The samples were then serially dehydrated in increasing concentrations of ethanol (ranging from 30% to 100%) to ensure complete dehydration. Finally, critical point drying was performed to completely dehydrate the samples before they were coated with platinum. SEM images were acquired on a ZEISS Gemini 450 scanning electron microscope using an accelerating voltage of 3.0 kV, a working distance of 6 mm, an Everhart-Thornley SE2 detector with an optically coupled photomultiplier, and the ZEISS SmartSEM (v. 6.05) software (49). The scale bars shown represent 200 nm.

### Sample preparation for LC-MS/MS analysis

A 100 µL of fungal EV solution (1 × 10^11^ p/mL) was used per sample. Samples were centrifuged at 13,500 × *g* for 20 min, and then 80 µL of the PBS was removed and replaced with 80 µL of sterile water. To prepare the fresh ethyl-acetate (EtOAc) solution, 10 mL of MilliQ water was added to 80 mL of EtOAc (LC-MS grade) and shaken three times with 5 min resting time in between. One milliliter of the upper EtOAc layer was used for the extraction. One mL of EtOAc solution was added to each sample and vigorously mixed for 1 min at room temperature (RT), then centrifuged at 16,000 × *g* for 15 s at RT, and the upper layer was aspirated. This was repeated six times. The remaining EtOAc was removed via speed vac. The samples were resuspended in 30 µL of solution comprising 2% acetonitrile (ACN) and 0.05% trifluoroacetic acid (TFA) and treated for 15 min in an ultrasonic bath. The sonicated sample was added to the filter (Millipore, Ultrafree-MC Hydrophilic PTFE membrane 0.2 µm), centrifuged at 14,000 rpm for 15 min and the flow through was transferred into high-performance liquid chromatography (HPLC) vials for subsequent LC-MS/MS analysis.

### Liquid chromatography coupled to tandem mass spectrometry analysis

LC-MS/MS analysis of fungal EVs was performed as described previously in Moyes et al. (15) with further optimization for the detection of candidalysin. Briefly, LC-MS/MS analysis was performed on an Ultimate 3,000 nano RSLC system (ThermoFisher Scientific) coupled to a QExactive Plus mass spectrometer (ThermoFisher Scientific). Peptide separation was performed at a flow rate of 300 nL/min on an Accucore C4 column (75 µm I.D. × 150 mm, 2.6 µm) with eluents (A) 0.2% HCOOH in 95:5 H_2_O-DMSO and (B) 0.2% HCOOH in 85:10:5 ACN-H_2_O-DMSO using the following gradient: 0–1.5 min at 60% B, 35–45 min at 96% B, and 45.1–60 min at 60% B. The top 15 precursor ions (full scan at m/z 350–1,500, R = 70 k [full width at half maximum, FWHM]) per scan cycle underwent high energy collisional dissociation fragmentation at 28% normalized collision energy. The resulting MS2 spectra were monitored at R = 17.5 k (FWHM). Proteome Discoverer 2.4 (ThermoFisher Scientific) and the Sequest HT algorithm were used to search against the protein database of *C. albicans* SC5314 (http://www.candidagenome.org). Mass spectra were searched for both unspecific cleavages (no enzyme) and tryptic peptides with up to four missed cleavages. Precursor and fragment mass tolerances were 10 ppm and 0.02 Da, respectively. At least two unique peptides per protein, a false discovery rate of <1%, and cross-correlation (Xcorr) validation (from 2.0 at z = 2 up to 3.0 at z = 6) were required for identification.

### Candidalysin peptide

Candidalysin (SIIGIIMGILGNIPQVIQIIMSIVKAFKGNK) was purchased from Peptide Protein Research Ltd. (UK). Candidalysin was synthesized using standard Fmoc chemistry and purified by HPLC to a minimum purity of 95%. Peptide purity and experimental molecular mass were further verified by LC-MS/MS. Candidalysin was dissolved in sterile water to a stock concentration of 3.0 mM. Stocks were aliquoted and stored at −20°C.

### Mammalian cell culture

All cell experiments were performed using the TR146 human OEC line (50), as described previously (18). OECs were cultured in a Dulbecco’s Modified Eagle Medium (DMEM)–F-12 medium nutrient mixture (1:1) plus L-glutamine (Life Technologies) supplemented with 10%–15% (vol/vol) heat-inactivated fetal bovine serum (FBS) (Life Technologies) in a humidified incubator at 37°C with 5% CO_2_. Except for the epithelial cell damage assay, 1% (vol/vol) penicillin-streptomycin (Sigma) was supplemented in a cell culture medium.

### Epithelial cell damage assay

To quantify cellular damage, 5 × 10^5^ TR146 cells/well were seeded in a 24-well format and cultured for 2 days in 500 µL of FBS-supplemented medium. Once confluent, the cells were washed with PBS, and a serum-free medium was added. EVs were added to epithelial cells in a concentration of 3 × 10^12^ particles/mL. Treated cells were cultured in a humidified incubator at 37°C with 5% CO_2_. Epithelial cell damage was analyzed 24 h post-treatment by measuring the activity of lactate dehydrogenase in sample supernatants using the Cytotoxicity Detection Kit (Roche) according to the manufacturer’s instructions.

### Treatment of epithelial cells with EVs and candidalysin

Prior to EV treatment, confluent OECs were serum starved overnight, and all experiments were performed in serum-free DMEM–F-12 medium. OECs were treated with EVs and candidalysin at the indicated concentrations for 2 h (western blotting) or 24 h (damage and cytokine assays). Treated cells were cultured in a humidified incubator at 37°C with 5% CO_2_.

### Quantification of secreted cytokines

After 24 h of EV (3 × 10^12^ p/mL) or candidalysin (7 and 15 µM) challenge, the exhausted culture medium was isolated. Human IL-1β, GM-CSF, and G-CSF (R&D Systems) were quantified by magnetic Luminex performance assay (Bio-Techne) and the Bio-Plex 200 system (Bio-Rad) according to the manufacturer’s instructions. Observed concentrations (pg/mL) were interpolated from a standard curve generated using beads with known concentrations for each target, calibrated on every run. This refers to the quantity of specific cytokines, derived from the fluorescence intensity emitted by bead-bound detection antibodies, expressed in pg/mL.

### Measurement of membrane permeability by EVs

To monitor membrane permeabilization induced by EVs, we reconstituted a horizontal DPhPC lipid bilayer in HEPES buffer pH 7.4 containing 100 mM KCl, using the painting technique across a small cavity on the MECA chip in Orbit e16 instrument (Nanion). MECA 16 chips contain 4 × 4 microcavities, providing a maximum of 16 horizontal DPhPC lipid bilayers for experimentation (26). Bilayers were stabilized for 15 min and their conductance was determined using a triangular waveguide. Intact bilayers with conductance values over 7 pF were used for further experiments. After incubating the bilayers at −50 mV for 10 min, only intact bilayers were used for monitoring EV-induced membrane permeabilization. EV samples at final concentrations of 1 × 10^12^, 3 × 10^12^, and 5 × 10^12^ p/mL were added to the bilayers and mixed by pipetting. Membrane permeabilization was monitored for 30 min at a constant applied potential of −50 mV.

### Kinetic analysis of EV-induced membrane permeabilization

To further analyze the membrane permeabilization properties of EVs, the latency from the addition of EV samples until membrane permeabilization was measured. Current data recorded by Orbit e16 were analyzed using Clampfit software 10.3. Average latencies with standard deviations were obtained from more than six independent replicates. The average latency values were compared between different fungal strains and different EV concentrations by one-way analysis of variance assay using Origin 2021 software.

### Detergent susceptibility using dynamic light scattering

To monitor detergent susceptibility, EVs were incubated for 30 min in PBS buffer with and without 0.1% Triton X-100, and the mean particle size was determined by DLS using a Protein Solutions DynaPro-MS/X system (Wyatt Technology Corporation, Santa Barbara, CA). The EVs were diluted to 3.75 × 10^10^ p/mL and transferred into a 96-well microplate (Nunc) (100 µL/well). After laser and temperature equilibration of the device, the 96-well plate was loaded into the device. The size of the EVs was determined via radial measurements. DLS measurements were performed with 10 acquisitions of 5 s and analyzed using DYNAMICS 8.1.1. software. Mean radius values and standard deviations were obtained from three independent replicates.

### Label-free gel-free biofilm EV proteomics

*C. albicans* biofilm EV proteomics analysis was performed as described elsewhere (40). EV samples were broken open with three volumes of a CHCl_3_:MeOH (2:1, vol/vol) mixture, vortexed for 30 s, and then subjected to centrifugation at 800 × *g* for 3 min. Both the aqueous and intermediate phases were collected, and the solvent was removed by drying in a Vacufuge 5305+ Concentrator Complete System (Eppendorf). Next, enzymatic “in liquid” digestion and mass spectrometric analysis were done at the Mass Spectrometry Facility, Biotechnology Center, University of Wisconsin–Madison. Two hundred micrograms of proteins were extracted by precipitation with 15% trichloroacetic acid/60% acetone and then incubated at −20°C for 30 min. The vesicle preparation was centrifuged at 16,000 × *g* for 10 min, and the resulting pellets were washed twice with ice-cold acetone, followed by an ice-cold MeOH wash. Pelleted proteins were resolubilized and denatured in 10 µL of 8 M urea in 100 mM NH_4_HCO_3_ for 10 min, then diluted to 60 µL for tryptic digestion with the following reagents: 3 µL of 25 mM DTT, 4.5 µL of acetonitrile, 36.2 µL of 25 mM NH_4_HCO_3_, 0.3 µL of 1 M Tris-HCl, and 6 µL of 100 ng/µL Trypsin Gold solution in 25 mM NH_4_HCO_3_ (Promega). Digestion was conducted in two stages, first overnight at 37°C, then an additional 4 µL of trypsin solution was added, and the mixture was incubated at 42°C for an additional 2 h. The reaction was terminated by acidification with 2.5% TFA to a final concentration of 0.3% and then centrifuged at 16,000 × g for 10 min. Trypsin-generated peptides were analyzed by nanoLC-MS/MS using the Agilent 1100 nanoflow system (Agilent) connected to a hybrid linear ion trap-orbitrap mass spectrometer (LTQ-Orbitrap, Thermo Fisher Scientific) equipped with a nanoelectrospray ion source. Capillary HPLC was performed using an in-house fabricated column with an integrated electrospray emitter. Sample loading and desalting were achieved using a trapping column in line with the autosampler (Zorbax 300SB-C18, 5 µm, 5 × 0.3 mm, Agilent). The LTQ-Orbitrap was set to acquire MS/MS spectra in a data-dependent mode as follows: MS survey scans from 300 to 2,000 m/z were collected in profile mode with a resolving power of 100,000. MS/MS spectra were collected on the five most abundant signals in each survey scan. Dynamic exclusion was employed to increase the dynamic range and maximize peptide identifications. Raw MS/MS data were searched against a concatenated *C. albicans* amino acid sequence database using an in-house MASCOT search engine30. Identified proteins were further annotated and filtered to 1.5% peptide and 0.1% protein false-discovery rate with Scaffold Q+ version 4.10.0 (Proteome Software Inc.) using the protein prophet algorithm. The mass spectrometry proteomics data have been deposited to the ProteomeXchange Consortium via the PRIDE partner repository with the data set identifier PXD045873 and 10.6019/PXD045873. Data are available via ProteomeXchange with the identifier PXD045873.

### Isolation and analysis of EV lipids

Lipid extraction from the desalted lyophilized EVs was carried out using a mixture of CHCl_3_/MeOH (2:1, vol/vol) along with 0.1 g/L of butylated hydroxytoluene (BHT). The mixture was vortexed, incubated in darkness at room temperature for 2 h, and subsequently subjected to centrifugation. The upper organic solvent layer was separated, leaving behind the pellet, which was then washed with 2 mL of CHCl_3_/MeOH (2:1, vol/vol) and subjected to another round of centrifugation. The gathered lipid extracts were combined and subjected to drying under a stream of nitrogen. Following the drying process, the sample was reconstituted using 0.5 mL of CHCl_3_/MeOH (2:1, vol/vol) and underwent TLC separation using 20 cm × 20 cm silica gel Si60 plates. Separation of neutral lipids took place using hexane/ethyl ether/AcOH (90:20:1, vol/vol), while polar lipids were separated using CHCl_3_/MeOH/AcOH/H_2_O (50:37.5:3.5:2, vol/vol). Subsequent visualization of lipids was achieved using UV light after spraying the plates evenly with a 0.2% fluorescein solution in EtOH. All extracted lipid classes were carefully scraped off the silica gel plates and re-extracted using CHCl_3_/MeOH (4:1, vol/vol) containing 0.1 g/L BHT. After vortexing, the samples were incubated overnight at room temperature and then subjected to centrifugation in order to remove any silica gel particles. To each sample, 100 µL of a 0.05 mg/mL pentadecanoic acid solution was added, followed by the evaporation of organic solvents using nitrogen gas. The isolated lipids then underwent methylation in the presence of 0.5 mL of 14% BF3 in MeOH. The processed lipid-containing vials were subjected to boiling, followed by mixing with 1 mL of hexane and 0.5 mL of H_2_O. After vortexing and centrifugation, the upper hexane layer containing methyl ester derivatives was carefully transferred to new clean glass tubes, dried using nitrogen gas, resuspended in 100 µL of hexane, and subsequently transferred to GC vials. Identification of fatty acid methyl esters was carried out using gas chromatography with a Shimadzu GC-2010 system equipped with a capillary column coated with DB-225 (30 m length, 0.25 mm internal diameter, 0.25 µm; Agilent Technologies, Inc.). Peaks were identified by comparing retention times with a set of authentic fatty acid standards provided by Supelco. The relative peak areas were utilized to calculate the abundance of fatty acids.

## SUPPLEMENTAL MATERIAL

Supplemental material iai.00404-24-s0001.docx Tables S1 to S4; Fig. S1.

ASM does not own the copyrights to Supplemental Material that may be linked to, or accessed through, an article. The authors have granted ASM a non-exclusive, world-wide license to publish the Supplemental Material files. Please contact the corresponding author directly for reuse.
