# Supplementary material for: Candida albicans biofilm extracellular vesicles deliver candidalysin to epithelial cell membranes and induce host cell responses
Source: Infect Immun. 2025 Apr 2;93(5):e00404-24. doi: 10.1128/iai.00404-24 (PMC12070735; doi:10.1128/iai.00404-24)
Supplement: Supplemental material — Tables S1 to S4; Fig. S1. [file iai.00404-24-s0001.docx]

**Supplementary Information**

**Supplementary Table 1. Nanoparticle tracking analysis of one of the EV batch #2.** Concentration and average diameter are shown.

| **EVs batch** | **Concentration (particles/mL)** | **Average diameter (nm)** |
| --- | --- | --- |
| Yeast | 1 x 10^14^ | 163.9 |
| 24 h biofilm | 3 x 10^13^, 4 x 10^13^ | 138.8 |
| 48 h biofilm | 3 x 10^13^ | 160.7 |

**Supplementary Table 2. Kinetic analysis of membrane permeabilization assay.** Latencies (seconds) of EV-induced membrane permeabilization are shown as a table with standard deviations (SD) and number of repeats.

|  | Latency (± SD) | Number of repeats |
| --- | --- | --- |
| WT-24 (1 x 10^12^ p/mL) | 430 (± 330) | 22 |
| WT-24 (3 x 10^12^ p/mL) | 210 (± 210) | 13 |
| WT-24 (5 x 10^12^ p/mL) | 120 (± 70) | 14 |
| WT-48 (1 x 10^12^ p/mL) | 1950 (± 570) | 10 |
| WT-48 (3 x 10^12^ p/mL) | 1360 (± 260) | 7 |
| WT-48 (5 x 10^12^ p/mL) | 420 (± 390) | 10 |
| *ece1*Δ/Δ-24 (1 x 10^12^ p/mL) | 1480 (± 760) | 10 |
| *ece1*Δ/Δ-24 (3 x 10^12^ p/mL) | 980 (± 370) | 8 |
| *ece1*Δ/Δ-24 (5 x 10^12^ p/mL) | 740 (± 360) | 6 |
| *ece1*Δ/Δ-48 (1 x 10^12^ p/mL) | 1480 (± 1470) | 10 |
| *ece1*Δ/Δ-48 (3 x 10^12^ p/mL) | 1090 (± 470) | 7 |
| *ece1*Δ/Δ-48 (5 x 10^12^ p/mL) | 1300 (±800) | 6 |

**Supplementary Table 3. Unique proteome present in WT EVs of 24 h in comparison with WT EVs of 48 h.**

| **From** | **Gene Names** | **Protein names** |
| --- | --- | --- |
| P43098 | FAS2 | Fatty acid synthase subunit alpha (EC 2.3.1.86) [Includes: Acyl carrier; 3-oxoacyl-[acyl-carrier-protein] reductase (EC 1.1.1.100) (Beta-ketoacyl reductase); 3-oxoacyl-[acyl-carrier-protein] synthase (EC 2.3.1.41) (Beta-ketoacyl synthase)] |
| Q5APD4 | MTS1 CFA1 CAALFM_C109680WA CaO19.12294 CaO19.4831 | Sphingolipid C9-methyltransferase (C-9-MT) (EC 2.1.1.317) (Cyclopropane fatty acyl phospholipid synthase homolog 1) (Methyltransferase for sphingolipid 1) |
| C4YTL2 | CAWG_05507 | Purine nucleoside permease |
| C4YS31 | CAWG_04889 | Uncharacterized protein |
| C4YIM2 | CAWG_04298 | ATP synthase subunit 4 |
| C4YGZ5 | CAWG_03336 | Reticulon-like protein |
| C4YNY8 | CAWG_02921 | Guanosine-diphosphatase |
| Q59V63 | EMP24 orf19.13672 CAALFM_CR07590WA | Emp24p |
| C4YFA9 | CAWG_01224 | arginine-tRNA ligase (EC 6.1.1.19) (Arginyl-tRNA synthetase) |
| Q59MJ6 | SEC61 CAALFM_C307810CA CaO19.6176 | Protein transport protein SEC61 subunit alpha |
| Q5ABS1 | QCR7 orf19.13074 CAALFM_C603400CA | Cytochrome b-c1 complex subunit 7 |
| C4YMF9 | CAWG_02039 | dihydrolipoyllysine-residue succinyltransferase (EC 2.3.1.61) |
| Q5AQ36 | SHO1 SSU81 CAALFM_C109140CA CaO19.12235 CaO19.4772 | High osmolarity signaling protein SHO1 (Osmosensor SHO1) |
| C4YTE2 | CAWG_05431 | Acyl-CoA-binding protein 2 |
| C4YE13 | CAWG_00763 | PRA1 family protein |
| Q5AI48 | CAALFM_C103120WA CaO19.10522 CaO19.3004 | FAS1 domain-containing protein CaO19.3004 |
| Q5A441 | orf19.8853 CAALFM_C405810WA | J domain-containing protein |
| C4YDW4 | CAWG_00712 | SCP domain-containing protein |
| C4YQH4 | CAWG_02732 | Uncharacterized protein |
| C4YRC9 | CAWG_04628 | Ecm33p |
| C4YPJ4 | CAWG_02395 | Hyphally-regulated cell wall protein N-terminal domain-containing protein |
| C4YIW9 | CAWG_04398 | valine--tRNA ligase (EC 6.1.1.9) (Valyl-tRNA synthetase) |
| C4YPZ9 | CAWG_02553 | glutamine--fructose-6-phosphate transaminase (isomerizing) (EC 2.6.1.16) (D-fructose-6-phosphate amidotransferase) (Hexosephosphate aminotransferase) |
| C4YF48 | CAWG_01159 | Importin beta-1 subunit |
| Q59US5 | MET15 orf19.13090 CAALFM_C400200CA | Bifunctional cysteine synthase/O-acetylhomoserine aminocarboxypropyltransferase |
| C4YTQ3 | CAWG_05548 | Mitochondrial import receptor subunit TOM40 |
| C4YQK4 | CAWG_02763 | Ribosomal protein L19 |
| Q59WF4 | MNN2 MNN25 MNN5 CAALFM_C110720CA CaO19.2347 CaO19.9883 | Alpha-1,2-mannosyltransferase MNN2 (EC 2.4.1.-) |
| C4YN06 | CAWG_02244 | D-arabinono-1,4-lactone oxidase (ALO) (EC 1.1.3.37) (L-galactono-gamma-lactone oxidase) |
| C4YMB9 | CAWG_01998 | C-1-tetrahydrofolate synthase, mitochondrial |
| C4YFL6 | CAWG_01334 | Sodium transport ATPase 2 |
| C4YF39 | CAWG_01150 | 60S ribosomal protein L26-B |
| C4YM47 | CAWG_01922 | Very-long-chain 3-oxoacyl-CoA reductase (EC 1.1.1.330) (3-ketoacyl-CoA reductase) (3-ketoreductase) (KAR) (Microsomal beta-keto-reductase) |
| C4YNU8 | CAWG_02881 | Glycine zipper 2TM domain-containing protein |
| C4YHJ3 | CAWG_03540 | Transmembrane 9 superfamily member |
| C4YS10 | CAWG_04868 | Dolichyl-diphosphooligosaccharide--protein glycosyltransferase subunit 3 |
| C4YDH3 | CAWG_00568 | Vacuolar calcium ion transporter |
| Q5A006 | orf19.11860 CAALFM_CR03600CA | Coatomer subunit zeta |
| C4YE92 | CAWG_00846 | 6,7-dimethyl-8-ribityllumazine synthase (DMRL synthase) (EC 2.5.1.78) |
| Q59WG0 | HNT1 orf19.2341 CAALFM_C110780CA | Adenosine 5'-monophosphoramidase HNT1 (EC 3.-.-.-) (Histidine triad nucleotide-binding protein HNT1) (HINT) |
| C4YLZ1 | CAWG_01864 | Protoplast secreted protein 2 |
| C4YKA7 | CAWG_05904 | D-arabinose dehydrogenase heavy chain |

**Supplementary Table 4. Comparative lipidomics of *C. albicans* biofilm EVs.** Values are nmol, signal per mg lipid WT. Data shown are the mean of n = 7 independent experiments.

|  |  | **24 h EVs** | | **48 h EVs** | |
| --- | --- | --- | --- | --- | --- |
| Lipid family | Lipid class | Mean | SD | Mean | SD |
| Polar | LysoPC | 0.815 | 0.371 | 1.090 | 0.674 |
| Polar | PC | 111.117 | 2.953 | 98.315 | 12.151 |
| Polar | LysoPE | 0.706 | 0.300 | 0.454 | 0.268 |
| Polar | PE | 25.136 | 3.196 | 24.866 | 8.849 |
| Polar | PI | 114.327 | 6.833 | 102.737 | 12.304 |
| Polar | PS | 13.584 | 2.378 | 12.387 | 6.024 |
| Polar | PA | 12.387 | 2.682 | 13.402 | 4.048 |
| Polar | PG | 5.047 | 1.304 | 3.758 | 1.750 |
| Sphingolipids | IPC | 1.799 | 0.319 | 1.355 | 0.440 |
| Sphingolipids | MIPC | 0.282 | 0.074 | 0.233 | 0.073 |
| Sphingolipids | MI(IP)2C | 0.039 | 0.022 | 0.048 | 0.033 |
| Neutral | ERG | 0.182 | 0.114 | 0.031 | 0.039 |
| Neutral | DAG | 17.849 | 2.636 | 9.453 | 1.234 |
| Neutral | TAG | 4.473 | 0.824 | 1.092 | 0.188 |


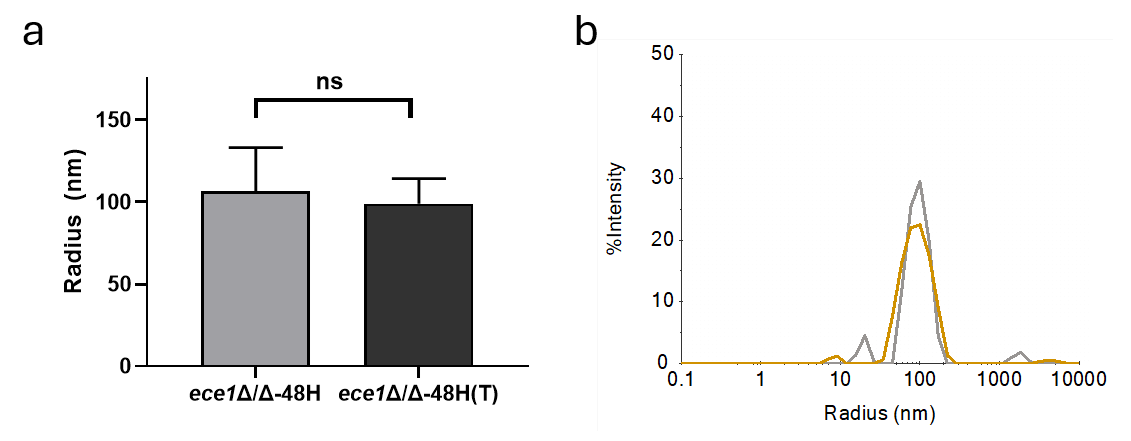


**Supplementary Figure 1. Candidalysin-deficient 48H EVs exhibit no differences in detergent susceptibility.** The radius of *ece1*Δ/Δ-48H EVs was monitored in response to treatment with Triton X-100 (T) using dynamic light scattering. **(a)** The bar chart represents the mean ± standard deviation of n = 5 technical repeats. **(b)** Hydrodynamic radius distribution by intensity for *ece1*Δ/Δ-48H EVs without (grey) and with Triton treatment (gold). The radius remained unchanged, indicating resistance to detergent-induced membrane rupture. Statistical analysis was performed using an unpaired two-tailed t-test (ns: non-significant).
